# Copy number of the *Adenomatous Polyposis Coli* gene is not always neutral in sporadic colorectal cancers with loss of heterozygosity for the gene

**DOI:** 10.1186/s12885-016-2243-z

**Published:** 2016-03-12

**Authors:** Peter Zauber, Stephen Marotta, Marlene Sabbath-Solitare

**Affiliations:** Department of Medicine, Saint Barnabas Medical Center, 22 Old Short Hills Road, Livingston, NJ 07039 USA; Department of Pathology, Saint Barnabas Medical Center, 100 Old Short Hills Road, Livingston, NJ 07039 USA

**Keywords:** Genetic mutations, Chromosomal number, Colon tumors, Loss of genetic material, *APC* gene, *KRAS* gene

## Abstract

**Background:**

Changes in the number of alleles of a chromosome may have an impact upon gene expression. Loss of heterozygosity (LOH) indicates that one allele of a gene has been lost, and knowing the exact copy number of the gene would indicate whether duplication of the remaining allele has occurred. We were interested to determine the copy number of the *Adenomatous Polyposis Coli* (*APC*) gene in sporadic colorectal cancers with LOH.

**Methods:**

We selected 38 carcinomas with LOH for the *APC* gene region of chromosome 5, as determined by amplification of the CA repeat region within the D5S346 loci. The copy number status of *APC* was ascertained using the SALSA® MLPA® P043-B1 APC Kit. LOH for the *DCC* gene, *KRAS* gene mutation, and microsatellite instability were also evaluated for each tumor, utilizing standard polymerase chain reaction methods.

**Results:**

No tumor demonstrated microsatellite instability. LOH of the *DCC* gene was also present in 33 of 36 (91.7 %) informative tumors. A *KRAS* gene mutation was present in 16 of the 38 (42.1 %) tumors. Twenty-four (63.2 %) of the tumors were copy number neutral, 10 (26.3 %) tumors demonstrated major loss, while two (5.3 %) showed partial loss. Two tumors (5.3 %) had copy number gain.

**Conclusions:**

Results of *APC* and *DCC* LOH, *KRAS* and microsatellite instability indicate our colorectal cancer cases were typical of sporadic cancers following the ‘chromosomal instability’ pathway. The majority of our colorectal carcinomas with LOH for *APC* gene are copy number neutral. However, one-third of our cases showed copy number loss, suggesting that duplication of the remaining allele is not required for the development of a colorectal carcinoma.

## Background

One of the molecular classifications of colorectal cancer (CRC) involves chromosomal instability. CRCs with chromosomal instability frequently demonstrate loss of heterozygosity (LOH) involving numerous genes, but particularly the *APC* (Adenomatous Polyposis Coli) and *DCC* (Deleted in Colorectal Cancer) genes, as well as point mutations in the *KRAS* (Ki-ras2 Kirsten rat sarcoma) gene. A second classification of CRCs involves microsatellite instability (MSI), involving short repetitive nucleotide sequences that become either shorter or longer than normal because of defective DNA repair mechanisms. CRCs with MSI generally do not have LOH of *APC* and *DCC*.

Loss of heterozygosity is deletion of genetic material involving one of the two (maternal or paternal) autosomal genes, or alleles. LOH is important in the process of carcinogenesis through at least two general mechanisms. Following the loss of chromosomal material of one allele, the remaining allele may be affected by a subsequent somatic mutation, thereby leaving no functional gene; or, the remaining allele may already contain a disease-prone mutation (germ line or somatic) that is now present in a hemizygous, and potentially more expressed, state [1]. Further, the loss of one allele may be followed by the duplication of the remaining allele through various mechanisms [2]. This is often referred to as acquired uniparental disomy, but it is perhaps more succinct to refer directly to the number of copies present. Even with duplication of the remaining allele, LOH is still present, as LOH refers to the loss of one parental gene, regardless of whether the remaining gene is single or duplicated.

Copy Number (CN) refers directly to the physical number of copies of a chromosome or a chromosomal region present in a cell. The normal number is two; but for tumor cells there may be more or less than two [3]. A change in CN results from a deletion or duplication of a length of DNA as compared with normal tissue, and CN changes may range in size from a kilobase to several megabases or even an entire chromosome, thereby involving one or more genes.

Reports of CN findings in colorectal cancer have evaluated multiple genes, and they do not specifically focus on the Adenomatous Polyposis Coli gene, which is a critically important gene in colorectal carcinogenesis. Further, the status of LOH for the tumors reported is not always clear. Limited data suggest that for colorectal tumors, half of the various LOH regions show no evidence of a reduction in DNA copy number, while a study of 94 sporadic colorectal cancers suggested that physical loss of chromosomal material is common [2, 4].

*APC* is a tumor suppressor gene located on the long (q) arm of chromosome 5 between positions 21 and 22. The clinical impact of germ line mutations in *APC* is exemplified by the development of hundreds to thousands of adenomas, as well as carcinomas, in patients with Familial Adenomatous Polyposis (FAP). Somatic mutations of *APC* occur early in the development of a sporadic colorectal carcinoma [5]. APC protein is an important part of the system controlling the level of *β*-catenin within the cytoplasm of cells. Failure to control cytosolic levels leads to an increase in nuclear β-catenin levels; where, in concert with another regulatory protein Wnt, it binds to transcription factors and facilitates tumorigenesis. Almost all colorectal tumors have a mutation in a key regulatory factor of the Wnt/*β*-catenin pathway, primarily the *APC* gene [6]. APC protein is also important in cell migration, adhesion, chromosome segregation, and spindle assembly [7].

The aim of our study is to focus on assessing CN for the *APC* gene in a well- defined group of colorectal cancers, each demonstrating significant LOH of *APC*. We hypothesized that definite LOH would clearly indicate one allele had been lost, and thus knowing the exact copy number of this critical gene would allow us to test the concept of allele duplication in sporadic colorectal cancer, and lead to a better understanding of the role of *APC* in colorectal carcinogenesis.

## Results

### Cases

There were 18 (47.4 %) females and 20 (52.6 %) males; the average age was 69.3 years with a range from 36 to 95 years. Thirteen (34.2 %) cancers were from the right colon and 25 (65.8 %) were from the left colon. Two cancers were adenocarcinomas with residual villous adenomatous tissue from which the carcinomatous portion was studied; one was stage 2 and one was stage 3. The other 36 cancers were invasive colorectal adenocarcinomas (Table 1).

**Table 1:** Clinical and molecular data for 38 colorectal adenocarcinomas with LOH of *APC* gene^a^

Tumor type	No.	%
Cancer	36	94.7
Cancer with Residual adenoma	2	5.3
Gender		
Male	20	52.6
Female	18	47.4
Location		
Right	1	2.6
Cecum	4	10.5
Ascending	5	13.1
Transverse	3	7.9
Descending	4	10.5
Sigmoid	18	47.4
Rectum	2	5.3
Left	1	2.6
*APC* LOH ratio		
Average	0.32	
Range	0.12–0.49	
*KRAS*		
Mutated	16	42.1
Wild type	22	57.9
*DCC*		
LOH	33	86.8
No LOH	3	7.9
Not done	2	5.3

^a^All cancers were microsatellite stable

### *APC* and *DCC* LOH, microsatellite instability and *KRAS* results

Cases were selected because they demonstrated definite LOH for the *APC* gene, as determined by one of the markers used. The average allele ratio for *APC* LOH was 0.32, with a range from 0.12 to 0.49. However, the ratio was less than 0.4 for 30 of the 38 (79 %) cases, indicating significant loss of one allele for the majority of cases. In addition to LOH of the APC gene, LOH of the *DCC* gene was present in 33 of 36 (91.7 %) informative tumors (Table 1), indicating the usual finding of consistency of LOH for these two genes, and providing further evidence that our cases represent typical CRC involving the ‘chromosomal instability’ pathway.

All cancers were microsatellite stable. *KRAS* mutations are also frequently detected in CRCs showing the ‘chromosomal instability’ pathway, and a *KRAS* gene mutation was present in 16 (42.1 %) of the 38 tumors. This percentage is consistent with the reported frequency of *KRAS* mutations in CRCs in general [8], indicating that our cases are typical sporadic CRCs. A total of 14 (87.5 %) of the *KRAS* mutations were in the second position of codons 12 or 13, and two were in the first position. A *KRAS* mutation was present in about half of the tumors that were copy number neutral, and in one-third of the tumors that showed copy number loss (data not shown).

### Multiplex ligation-dependent probe amplification results

**Fig. 1:**
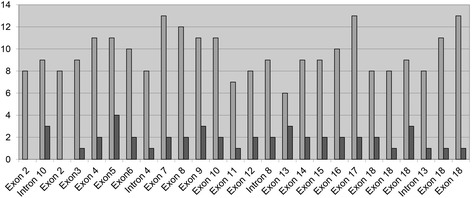
Gains (*black*) and losses (*light grey*) for 26 *APC* gene probes as determined by analysis of 38 colorectal carcinomas using SALSA® MLPA® P043-B1 Kit. The *APC* location is shown on the horizontal axis and the number of gains or losses on the vertical axis

It is of interest to first consider the number of losses and gains for each of the 26 *APC* MLPA (multiplex ligation-dependent probe amplification) probes used to study the carcinomas (Fig. 1). All probes demonstrated some losses, as indicated by the light grey vertical bars. Twenty-four of the 26 (92.3 %) probes demonstrated gains, as indicated by the black bars. Losses exceeded gains for each probe. The number of losses and gains for each probe is fairly consistent for all 26 probes. This indicates that no *APC* region presents as a ‘hot spot’ for gain or loss.

### Copy number results

**Fig. 2:**
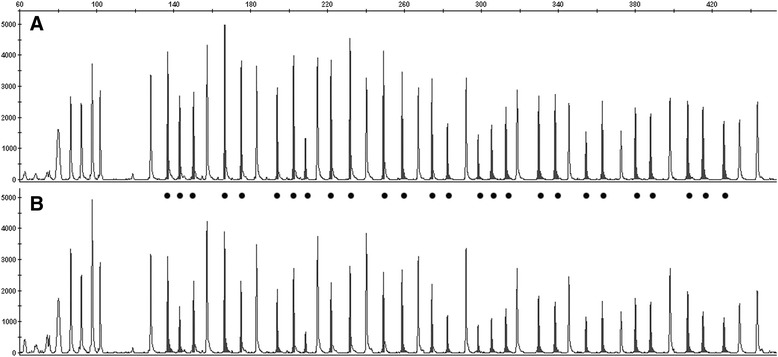
Capillary electrophoresis pattern for *APC* copy number. The Y-axis is the fluorescence signal intensity and the X-axis is the fragment size in base pairs, extending from 60 to 420 (indicated across the top). The peaks below 110 base pairs are quality control fragments. The 26 peaks highlighted by black circles are *APC* gene probe fragments, and the remaining 13 unhighlighted peaks are reference probe fragments. **a**. DNA from normal colon tissue. **b** DNA from colon carcinoma from the same patient showing diminished intensity with all *APC* probe fragments, relative to the normal tissue peaks

Twenty-four (63.2 %) of the 38 cancers were copy number neutral, and 14 (36.8 %) were not copy number neutral, at the loci of the *APC* gene studied. Two of these 14 (5.3 % of the entire cohort) demonstrated partial loss, with, respectively, 12 and 13 (out of 26) exons showing loss. Ten other tumors (26.3 % of the entire cohort) demonstrated major loss of at least this segment of the allele, with more than 13 exons showing loss. An example of data generated using multiplex ligation-dependent probe amplification for one tumor with copy number loss is shown in Fig. 2. There was no statistical difference in the *APC* gene alleles ratio between those tumors with copy number loss (APC allele ratio = 0.30) and those tumors that were copy number neutral (*APC* allele ratio = 0.33), with *p* = 0.35 by T-test. Two tumors (5.3 % of the entire cohort) demonstrated gain, with, respectively, 16 and 25 exons showing gain. *KRAS* mutational status also had no relationship to copy number results (*p* = 0.72) (Table 2).

**Table 2:** Copy number (CN) data of 38 colorectal cancers with LOH of *APC* gene

			Average no. of exons with:	
CN finding	No.	%	Gains	Losses
Neutral	24	63.2	0.3	0.8
Loss	10	26.3	0	20.5
Partial loss	2	5.3	0	12.0
Gain	2	5.3	19.5	0.5

For patients less than or equal to 60 years of age, 8 (80 %) had copy neutral tumors and 2 (20 %) had tumors showing copy number loss. For those over age 60 years, 16 (61.5 %) patients had copy number neutral tumors and 10 (38.5 %) had tumors with loss. This was not significantly different, with *p* = 0.44 (Fisher exact, 2-sided). Among male patients, 10 (55.6 %) had copy number neutral tumors and 8 (44.4 %) had tumors with copy number loss; while among female patients, 14 (77.8 %) had tumors that were copy number neutral and 4 (22.2 %) had tumors with copy number loss. This was not significant, (*p* = 0.29). For patients with left-sided colon tumors, 17 (70.8 %) were copy number neutral and 7 (29.2 %) showed loss; while for right-sided tumors, 7 (58.3 %) were copy number neutral and 5 (41.7 %) showed loss. This was not significantly different, (*p* = 0.48).

## Discussion

Numerous studies have shown that LOH of genetic material is deleterious when present either in the germ line [9, 10] or as a somatic development [11]. Several different mechanisms have been described that might contribute to the development of LOH [12]. If part, or all of one of the two alleles of a chromosome is lost, then an additional issue is whether the remaining allele is present singly or is copied once, thereby renewing the diploid status, referred to as ‘copy number neutral’. Doubling of the remaining allele will amplify any genetic alteration present on the template allele, and possibly augment its negative effect.

Studies of copy number in colorectal cancer have primarily reported genome-wide findings [4, 13, 14]. In particular, there are very few studies addressing both *APC* LOH and copy number. Sieber et.al. studied a mixture of adenomas and carcinomas from patients with attenuated Familial Adenomatous Polyposis (aFAP) and reported normal copy number in 17 tumors with LOH [15]. Jones et. al. studied large colorectal adenomas from patients with FAP, MYH-associated polyposis, as well as isolated adenoma cases. One adenoma was found to have LOH for the *APC* gene and was copy number neutral [16]. Melcher et. al. studied nine microsatellite stable colorectal cancer cell lines and two demonstrated homozygosity for the *APC* gene region and were copy number neutral [17]. Similar results regarding *APC* from a study of colon cancer cell lines were also reported by Segditas et al. [18].

Whether or not a single, remaining, allele is copied to produce copy number neutrality would involve DNA replication systems within the cell. We did not specifically study this issue; nor are we aware of any information from the literature indicating why replication occurs in some situations but not in others. It could depend on whether duplication of the remaining allele is, or is not, beneficial to the tumor cell.

Overall, approximately two-thirds of our cases were copy number neutral, indicating that the majority of colorectal cancers in which one of the two *APC* alleles is lost, do in fact, undergo duplication of the remaining allele. However, one-third of our colorectal cancers showed partial or complete loss of one of the *APC* alleles.

Our findings raise the additional question of what effect *APC* copy number has upon the carcinogenesis process. Our study does not directly address this, and there is no clinical correlation in this regard concerning *APC* in the literature. However, genomic amplification of other oncogenes has been associated with adenoma recurrence and the presence of synchronous carcinomas [19], lymph node metastases [2], and prognosis [14].

If the allele that is lost carried a normal *APC* gene, and the remaining *APC* gene contained a significant mutation, the result would be complete loss of APC protein function, and duplication of the mutated gene might not additionally facilitate carcinogenesis. However, there is the possibility that an *APC* mutation might be more harmful as a double, rather than a single allele, with respect to some of the many other functions of APC protein. Furthermore, there could be an impact from other mutated linked genes whose carcinogenic effects may be augmented by the duplication of the remaining allele. It is possible that the molecular ‘housekeeping’ process of maintaining copy number neutrality may be functioning adequately, whether or not there is an *APC* gene mutation in the remaining allele, and given sufficient time between allelic loss and surgical tumor removal, there will be an attempt to restore copy number neutrality. Copy number gain, where the number of alleles is greater than two, might reflect an abnormality in the usual housekeeping process, resulting in several duplications.

## Conclusion

We have demonstrated that the CRCs we studied had a typical molecular prolife for the ‘chromosomal instability’ pathway. We have further shown that most of these sporadic colorectal carcinomas with LOH for *APC* do return to copy number neutrality. However, some failed to return to *APC* gene copy number neutrality, suggesting that duplication of the remaining allele is not always required for the development of a colorectal carcinoma.

## Methods

We have completed several studies that evaluated particular groups of patients with colorectal neoplasms, with respect to molecular genetic abnormalities, both germ line and somatic [20, 21]. With each of these studies, patients were referred for participation by endoscopists, or they were self-referred. After being provided details of the study, patients signed a consent form agreeing to analyses of their banked tissue. Each individual study was approved by the Saint Barnabas Medical Center Institutional Review Board. This current analysis utilized cancers from patients who, by clinical and pathology records, clearly did not have Familial Adenomatous Polyposis (FAP) or hereditary nonpolyposis colorectal cancer. FAP is an inherited disorder primarily characterized by the development of colorectal polyps beginning at a young age, and numbering in the hundreds or thousands, leading to the development of CRC. HNPCC is an inherited disorder of DNA mismatch repair, resulting in a high risk for the development of CRC as well as other cancers. MSI is the hallmark of these tumors. All cancers for this study demonstrated definite loss of heterozygosity for the *APC* gene.

All colorectal cancer cases were archived material from our Department of Pathology, which processes approximately 25,000 surgical samples yearly. Clinical material primarily reflects a suburban community of middle economic level, with substantial representations from various minority groups (Asian, African-American). Histological slides stained with hematoxylin and eosin (H&E) were available for all cases, as were paraffin blocks containing adequate material to ensure sufficient DNA for analysis. One clinical pathologist reviewed all histological slides and indicated the areas for molecular study. All carcinomas were studied prior to the administration of any radiation or chemotherapy to the patient. Family and personal history was available from hospital records and from telephone interviews. The Saint Barnabas Medical Center Institutional Review Board approved this specific study.

### DNA extraction and purification

All tissue specimens were formalin-fixed and paraffin-embedded. Histological slides stained with H&E were examined and the areas of relevant tissue were identified and marked, as was an area of normal tissue. Consecutive unstained slides were prepared from the paraffin blocks and the corresponding areas were isolated with a blade under a dissecting microscope and transferred to an eppendorf tube. The paraffin wax was removed with xylene and ethanol washes. The cellular material was lysed in a proteinase K buffer solution. DNA was isolated and purified using the Qiagen QIAamp DNA Mini Kit (Qiagen Inc., Valencia, CA). DNA concentration was determined using a NanoDrop ND-1000 spectrophotometer (NanoDrop Technologies, Wilmington DE).

### Microsatellite analysis for loss of heterozygosity and microsatellite instability

All microsatellite primer sets were ordered through the Life Technologies Custom Oligo Synthesis Service (genomicorders@lifetech.com). In all primer sets the forward primer contained a 5′ fluorescent label while the reverse primer contained a 5′- GTGTCTT tail. All PCR reactions were performed in 30 μl volumes using 100 ng of template with Applied Biosystems reagents (Roche Molecular Systems, Inc., Branchburg, NJ) and a final 1.5 mM MgCl_2_ concentration. Reactions were run on an ABI 9700 thermal cycler (Applied Biosystems, Foster City, CA) under the following conditions: 5 min denaturation at 94 °C, followed by 35 cycles of a 30 s denaturation at 94 °C, 30 s annealing at 55 °C, and a 60 s elongation at 72 °C, with a final 30 min extension at 72 °C. PCR products were separated by capillary electrophoresis with an ABI 3130 Genetic Analyzer and the data was processed with GeneMapper software v4.0 (Applied Biosystems, Foster City, CA).

Loss of heterozygosity of the *APC* gene was determined by amplification of the CA repeat region within the D5S346 loci. PCR of microsatellites is a standard method for determining LOH. D5S346 demonstrates linkage disequilibrium with *APC* and is thus a well suited marker. The primer sequences used were: 5′-ACT CAC TCT AGT GAT AAA TCG GG-3′ (forward) and 5′-AGC AGA TAA GAC AAG TAT TAC TAG TT-3′ (reverse). Samples that were homozygous using the D5S346 primer set were analyzed using repeats within the D5S1965 or D5S492 loci. LOH of the *DCC* gene was determined by amplification of the CA repeat marker D18S1407 with the primers 5′-TTC CCT TCA TTT CAC TGG GA-3′ (forward) and 5′-CTA GAT GGA TGT GAC TTG GC-3′ (reverse). Samples that were homozygous using the D18S1407 primer set were analyzed using repeats within the D18S58 or D18S61 loci. For all LOH studies, neoplastic tissue was evaluated simultaneously with normal colonic mucosal tissue from the same patient.

**Fig. 3:**
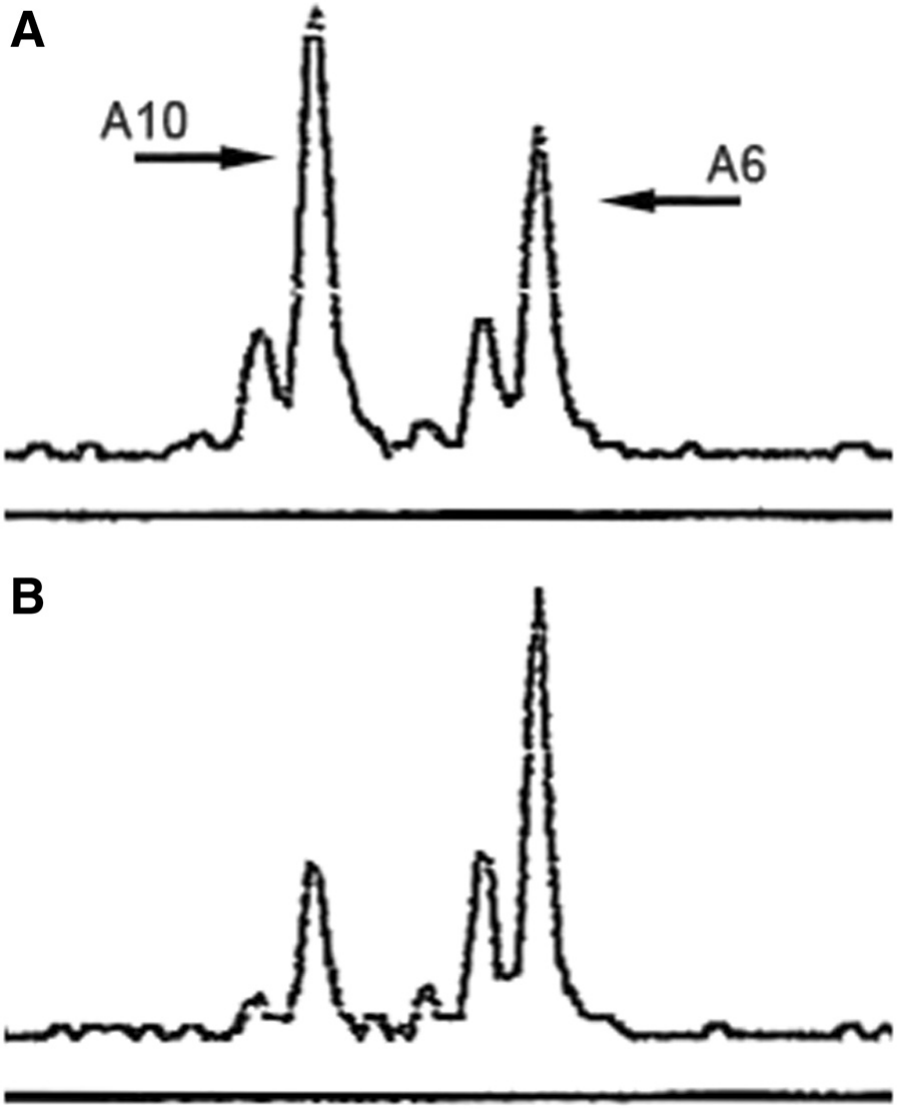
Electropherogram showing D5S346 microsatellite analysis of DNA used to determine loss of heterozygosity of *APC*. **a** Normal mucosa indicating the patient’s two alleles, A6 and A10. **b** Ascending colon carcinoma. The A10 allele is diminished, resulting in significant reversal of the relative intensities of the two bands

The primers used for LOH analysis all contain polymorphic CA repeats that usually generate two distinct PCR allele products (maternal and paternal). However, if the alleles are the same molecular weight, then they coincide. That particular CA repeat is considered homozygous and the primer set is uninformative for that sample. If the alleles are of different molecular weights, then the ratio of the allele peak heights is used to determine LOH. We define the allele ratio as the height of the higher molecular weight PCR product to the lower molecular weigh PCR product. In normal tissue, the lower molecular weight allele PCR product is always greater in height than the higher molecular weight product due to normal PCR bias (Fig. 3). Therefore, to determine LOH, the ratio of the band intensities (peak heights) of the two alleles in the neoplastic tissue is divided by the corresponding ratio in the normal mucosa. This ratio of ratios normalizes for this intrinsic PCR amplification bias, as well as for any inter-run variations in absolute peak heights. Resultant ratios of tumor to normal tissues that approach 1 are considered to have no LOH. We define a tumor to have LOH if the resultant ratio is less than 0.5 or greater than 2.0, depending on whether the higher or lower molecular weight allele is lost, respectively. This is a conservative value, but one that insures true LOH [22]. Approximately 40 % of the 530 colorectal cancers we have studied demonstrated LOH of the *APC* gene by our criteria. We selected 38 cases with sufficient available DNA and with an *APC* allele ratio below 0.5, with an average of 0.32 and a range from 0.12 to 0.49 (Table 1).

Microsatellite instability (MSI) was detected using the Bethesda panel of markers that includes two mononucleotide markers: BAT25 and BAT26, and three dinucleotide markers: D2S123, D5S346, and D17S250. A 1997 National Cancer Institute workshop recommended a ‘reference panel’ of 5 microsatellite markers for the detection of MSI in colorectal cancer [23]. This panel continues to be a useful screen for MSI. For all MSI studies neoplastic tissue was evaluated simultaneously with normal colonic mucosal tissue from the same patient. Microsatellite instability for a given primer set was defined as a change in the allele pattern, with the appearance of one or more new PCR products relative to those produced by the normal DNA. A tumor was defined as MSI-high if two or more of the five markers had a changed allele pattern, and is referred to as “MSI”. Since all tumors were microsatellite stable, we concluded that the MSI pathway was not a part of the molecular profile in these cases, and we did not assay for methylation.

### Sequence analysis of the *KRAS* gene

We used Sanger sequencing for detecting *KRAS* gene point mutations. Sanger sequencing is the gold standard for determining point mutations. The codon 12/13 region in exon 2 of the *KRAS* oncogene was amplified using the primer set 5′-AAGGCCTGCTGAAAATGACTG-3′ and 5′-GGTCCTGCACCAGTAATATGCA-3′. Hot-start PCR was performed in 50 μl volumes with AmpliTaq Gold polymerase and ABI reagents using 100 ng of template DNA, 50 pmols of primer, and 2.0 mM MgCl_2_ on a GeneAmp PCR System 9700 (Applied Biosystems, Foster City, CA). PCR consisted of an initial 8 min denaturation at 94 °C, followed by 40 total cycles of a 30 s denaturation at 94 °C, a 30 s annealing, and a one minute elongation at 72 °C, with a final 30 min extension at 72 °C. The annealing temperature was stepped down at 62, 60 and 58 °C for 5, 20, and 15 cycles, respectively.

The post-PCR products were quality checked by agarose gel and then purified using the QIAquick PCR Purification Kit (Qiagen Inc., Valencia, CA) prior to sequencing. The sequencing reactions were performed in 20 μl volumes using 0.5X BigDye Terminator Cycle Sequencing Reagents (Applied Biosystems, Foster City, CA), 5.0 pmol of the reverse *KRAS* primer, and 1.0 μl of the purified PCR reaction. Reactions were run on a GeneAmp PCR System 9700 for 25 cycles using a 2-minute extension time. The sequencing reaction fragments were cleaned using isopropanol precipitation. Sequencing products were separated by capillary electrophoresis with an ABI 3130 Genetic Analyzer and the data was processed with Sequencing Analysis v5.2 software (Applied Biosystems, Foster City, CA).

### Copy number analysis of the *APC* gene

The copy number status of the *APC* gene was ascertained using multiplex ligation-dependent probe amplification [24]. We employed the SALSA® MLPA® P043-B1 APC Kit according to the manufacturer’s instructions (MRC-Holland, Amsterdam, The Netherlands). We used this method because the MLPA method allows amplification of multiple PCR targets with a small amount of DNA harvested from paraffin blocks.

This kit uses 26 probes spanning the APC gene and additional 13 reference probes. Tumor and normal DNA from paraffin-embedded tissue was analyzed as sample and reference runs, respectively. Two hundred nanograms of genomic DNA were hybridized with the probe mixture followed by a ligation reaction. All of the probes contain universal PCR primer recognition sites, and only ligated probes were subsequently PCR amplified. A single PCR reaction therefore amplified all 39 ligation products. PCR reactions were analyzed on an ABI 3130 Genetic Analyzer with GeneMapper v4.0 software. GeneMapper data were subsequently exported for copy number analysis using Coffalyser v9.4 software available at the MRC-Holland website (www.mlpa.com).

Within the Coffalyser software, the “Tumor Analysis Least Squares (LS)” analysis option was chosen to normalize and analyze the MLPA data. This method normalizes (intra-sample) the *APC* probe peak areas versus the reference probes areas for all sample runs, and then normalizes (inter-sample) each tumor specimen run versus the normal DNA reference runs. The ratios of the changes in the APC probe areas from the tumor DNA verses the normal reference DNA are reported. The Coffalyser analysis assigns ratio values less than 0.7 as having a copy number loss, greater than 1.3 as a having a copy number gain, and ratio values between 0.7 and 1.3 as normal for each *APC* probe [25]. This assay does not determine exact number of copies of *APC*, rather it provides information relative to normal tissue and to the internal control.

For any given tumor sample, we assigned overall copy number loss or gain if 2/3 (rounded to the nearest whole number) or more of the 26 APC probes had ratio values consistently <0.7 or >1.3, respectively. If 1/3 (rounded to the nearest whole number) or less or the 26 APC probes showed a loss or gain we assigned the sample as copy number neutral. If 1/3 to 2/3 of the probes showed a consistent loss or gain we assigned the sample as having a partial copy number loss or gain.

### Statistical methods

The t-test was used to assess whether the *APC* ratio differed by copy number status (neutral or loss). The Fisher’s exact test (two-sided) was used to assess whether copy number status differed by age (<=60, >60), sex, or location (proximal or distal) in the colon.

## Abbreviations

LOH: loss of heterozygosity
APC gene: adenomatous polyposis coli gene
DCC gene: deleted in colorectal cancer gene
KRAS gene: Ki-ras2 Kirsten rat sarcoma gene
MSI: microsatellite instability
MLPA: multiplex ligation-dependent probe amplification
FAP: familial adenomatous polyposis
MYH gene: mutY homolog gene
H&E: hematoxylin and eosin
